# Environmental Modeling, Technology, and Communication for Land Falling Tropical Cyclone/Hurricane Prediction

**DOI:** 10.3390/ijerph7051937

**Published:** 2010-04-28

**Authors:** Francis Tuluri, R. Suseela Reddy, Y. Anjaneyulu, John Colonias, Paul Tchounwou

**Affiliations:** College of Science, Engineering and Technology, Jackson State University, 1400 Lynch Street, MS 39219, USA; E-Mails: remata.s.reddy@jsums.edu (R.S.R.); yerramilli.anjaneyulu@jsums.edu (A.Y.); john.colonias@jsums.edu (J.C.); paul.b.tchounwou@jsums.edu (P.T.)

**Keywords:** environmental modeling, tropical cyclone/hurricane prediction and communication

## Abstract

Katrina (a tropical cyclone/hurricane) began to strengthen reaching a Category 5 storm on 28th August, 2005 and its winds reached peak intensity of 175 mph and pressure levels as low as 902 mb. Katrina eventually weakened to a category 3 storm and made a landfall in Plaquemines Parish, Louisiana, Gulf of Mexico, south of Buras on 29th August 2005. We investigate the time series intensity change of the hurricane Katrina using environmental modeling and technology tools to develop an early and advanced warning and prediction system. Environmental Mesoscale Model (Weather Research Forecast, WRF) simulations are used for prediction of intensity change and track of the hurricane Katrina. The model is run on a doubly nested domain centered over the central Gulf of Mexico, with grid spacing of 90 km and 30 km for 6 h periods, from August 28th to August 30th. The model results are in good agreement with the observations suggesting that the model is capable of simulating the surface features, intensity change and track and precipitation associated with hurricane Katrina. We computed the maximum vertical velocities (W_max_) using Convective Available Kinetic Energy (CAPE) obtained at the equilibrium level (EL), from atmospheric soundings over the Gulf Coast stations during the hurricane land falling for the period August 21–30, 2005. The large vertical atmospheric motions associated with the land falling hurricane Katrina produced severe weather including thunderstorms and tornadoes 2–3 days before landfall. The environmental modeling simulations in combination with sounding data show that the tools may be used as an advanced prediction and communication system (APCS) for land falling tropical cyclones/hurricanes.

## 1. Introduction

Over the last decade, there has been an overall increase in the number of Atlantic hurricanes and those making landfall in the United States [1]. The 2005 hurricane season serves as a prime example with 27 named systems, three Category 5 hurricanes and unprecedented loss of life (>1,000 fatalities) and damage (>$100 billion) in the United States. Severe weather is an unusual circumstance that has the potential for significant destruction and loss of life. Thunderstorms, tornadoes, and hurricanes are commonly associated with severe weather. Each year, on an average, ten tropical storms (of which six become hurricanes) develop over the Atlantic Ocean, Caribbean Sea, or Gulf of Mexico. However, the formation region of most hurricanes that affect the United States is the Gulf of Mexico or Atlantic Ocean. The hurricane season is generally during June–November.

Hurricanes usually develop over the warm ocean waters with sea surface temperatures (SST) exceeding 26 °C. Hurricanes get their energy from evaporation over large expanse of warm tropical water. Evaporation from warm sea surface produces water vapor that condenses and releases latent heat, which intensifies the storm. The formation region of most hurricanes that affect the United States is the Atlantic Ocean, including the Gulf of Mexico. The region of formation is from 5° to 30° north and south of latitudes. This region is not only characterized by warm ocean temperatures, but also has sufficient Coriolis acceleration to cause the storm to rotate. Therefore, sea surface temperature is considered a major force behind the development of a hurricane. Ocean-atmospheric interactions play a prevalent role in exchanging heat, momentum and moisture fluxes [2,3]. The structure of a matured hurricane consists of spiral bands of individual convective cells organized into regions of rising and sinking air parcels, associated with small scale cumulus convection [4,5].

During the development of storms, the convective available potential energy or CAPE, is used as an index for large scale disturbances leading to severe weather. CAPE is a measure of the amount of buoyant energy that would be available to speed up air parcel vertically and can be used to estimate the maximum vertical speed [4,5]. Therefore, high CAPE values are required for the development of thunder storms, and higher the CAPE the more energy would be available for the growth of the storm. A CAPE of about 2,500 J/kg may give rise to an environment to trigger a moderately unstable atmosphere. Numerical modeling investigations have been used to simulate thunderstorms for understanding various dynamical and physical processes taking place within them [6,7], though earlier numerical simulations suggest that CAPE has less significance on hurricane intensity [6], but the signatures of the intensifying storm from CAPE values may be used for assessing pre-land fall effects to formulate an early warning and prediction of the situation.

The history of hurricanes reveals large scale disaster that they can cause in terms of human life, property damage on top of the disruption of normal life. In particular, the damage hurricane Katrina caused is a reminder for preparedness of the people, and demands the need to developing an early warning and advanced prediction and communication system (APCS). Human tendency does not give greater attention to long term preparation required of the effects of natural disasters more than on the disaster mitigation management. Hurricane Katrina has initiated the concept of preparedness to natural disasters.

**Figure 1.**
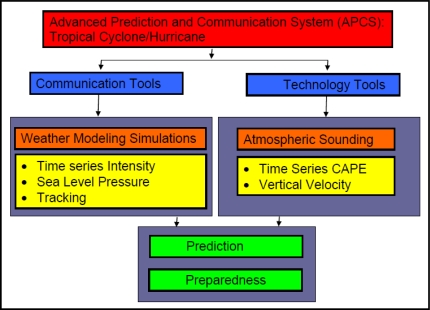
The proposed scheme of Advanced Prediction and Communication System (APCS) for Tropical Cyclone/Hurricanes.

In the present study, we propose a model of APCS as shown in Figure 1 by combining environmental modeling (WRF model) and atmospheric sounding data. The weather modeling simulations will be used to obtain hurricane intensity change, sea level pressure, precipitation, and tracking. The atmospheric soundings provide information on large scale convective instability of the atmosphere, CAPE, and maximum vertical velocities [4,5,8]. The results of the two techniques will be used to see signatures of pre-land fall effects during hurricane land-falling. An early warning prediction and preparedness will give a better crisis planning and emergency management that may reduce loss of life, property, and revenue in the event of high flood surge and inundation apart from others.

## 2. Experimental Section

### 2.1. History of Hurricane Katrina

The storm developed as an inner core that evolved into a deeper cyclone on 24 August 2005, and came under the influence of a strengthening middle to upper troposphere ridge over the northern Gulf of Mexico and southern United States. This ridge turned Katrina westward on 25 August toward southern Florida. Katrina generated an intense burst of deep convection over the low-level center during the afternoon of 25 August while positioned over the northwestern Bahamas. Further strengthening ensued, and Katrina is estimated to have reached hurricane status on 25 August 2005 at around 2100 UTC, less than two hours before its center made landfall on the southeastern coast of Florida [9,10].

### 2.2. Mesoscale Modeling

**Figure 2.**
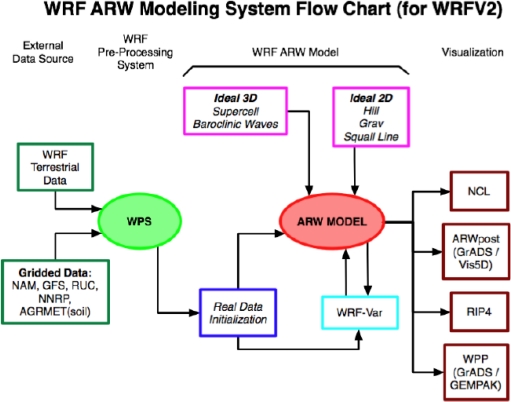
Flowchart for the WRF Modeling System, and the major Program Components consists of (1) The WRF Preprocessing System (WPS), (2) WRF-Var, (3) ARW Model solver, and (4) Post-processing graphics tools. The program units are described in the text, and details are available in the user guide [12].

The surface characteristics of hurricane Katrina were simulated using NCAR Weather Research Forecast (WRF) model (Figure 2). The model is based on fully compressible non-hydrostatic equations and the prognostic variables include the three dimensional wind, perturbation quantities of potential temperature, geo-potential, surface pressure, turbulent kinetic energy and scalars such as water vapor mixing ratio, cloud water. The details of Advanced Research WRF (ARW) model are described by Skamarock *et al.* [11] and also available for the users at the UCAR public domain website [12,13].

#### 2.2.1. Description of the flow chart

As described in the WRF user guide [11–13], the WRF Modeling System consists of four major programs: (1) The WRF Preprocessing System (WPS), (2) WRF-Var, (3) ARW solver, and (4) Post-processing graphics tools.

1. WPS: The WPS program is used primarily for real-data simulations. Its major functions include: (1) defining simulation domains; (2) interpolating terrestrial data (such as terrain, landuse, and soil types) to the simulation domain; and (3) degribbing and interpolating meteorological data from another model to this simulation domain. WPS takes the WRF terrestrial data/Gridded data as the input, and its output goes to the real data initialization.
2. WRF-Var: The WRF-Var program is used to ingest observations into the interpolated analyses created by WPS. It can also be used to update WRF model's initial condition when WRF model is run in cycling mode. This program is optional, and is not used in the present work.
3. ARW Solver: The unit is the key component in the modeling system, and is composed of several initialization programs for idealized, and real-data simulations, and the numerical integration program. ARW Model Solver takes the input from the initialization unit, and the model output is given to the visualization program. The model is fully compressible nonhydrostatic equations with hydrostatic option on a staggered Arakawa C grid. The wind components u, v, and w are recorded at the respective cell interfaces. The vertical velocity coordinate is a terrain following hydrostatic pressure coordinate. The solver uses the Runge-Kutta 3rd order time integration scheme and 5th order advection options along horizontal direction and the 3rd order in vertical direction. The other key features of the WRF model include:
  - complete coriolis and curvature terms
  - two-way nesting with multiple nests and nest levels
  - map-scale factors for conformal projections:
    ○ polar stereographic
    ○ Lambert-conformal
    ○ Mercator
  - time-split small step for acoustic and gravity-wave modes:
    ○ small step horizontally explicit, vertically implicit
    ○ divergence damping option and vertical time off-centering
    ○ external-mode filtering option
  - lateral boundary conditions
    ○ idealized cases: periodic, symmetric, and open radiative
    ○ real cases: specified with relaxation zone
  - full physics options for land-surface, PBL, radiation, microphysics and cumulus parameterization
  - grid analysis nudging and observation nudging
4. Graphics Tools: Several programs are supported, including RIP4 (based on NCAR Graphics), NCAR Graphics Command Language (NCL), and conversion programs for other readily available graphics packages: GrADS and Vis5D. Graphic Tools facilitate visualization of the model output and we have used GrADS in the present work. The details of these programs are described in the chapters of the user’s guide [12].

#### 2.2.2. ARW model configuration

**Figure 3.**
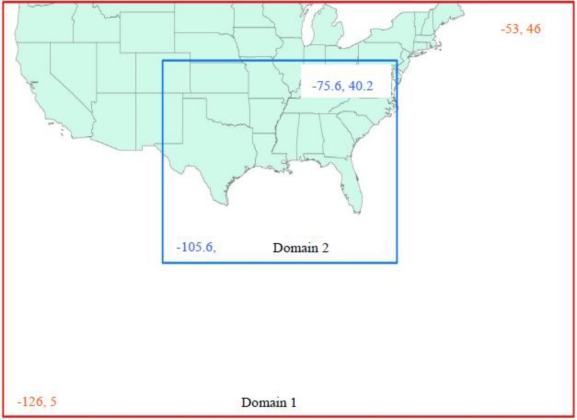
WRF model doubly nested domains fixed over the Gulf of Mexico and Florida region. Two domains of dimensions 81 × 47 and 100 × 67 are used with horizontal grid spacing of 90 km and 30 km respectively.

The ARW model with data assimilation was run for a period of three days, August 28–30, 2005 for six hour periods. Two domains of dimensions 81 × 47 and 100 × 67 are used for simulation over the Gulf of Mexico. The doubly nested domains used for the model runs are shown in Figure 3. Horizontal grid spacing of 90 km and 30 km are fixed over the Gulf of Mexico and Florida region at central latitude of 30.2N and longitude of 89.6W. Real Data was taken from UCAR’s NCEP Global Analyses for initial and lateral boundary conditions. We have noted from the previous studies that best agreement with the observations is obtained with the following schemes of different physical processes: Simple ice mixing (Microphysics), Blackadar planetary boundary layer parameterization, Cloud-resolving radiation, Grell cumulus parameterization [14–22]. The various options of the physics and grid configuration used in running the ARW model is given in Table 1.

For the purpose of model validation, the surface characteristics will be compared with the observed data provided by NOAA [9,10]. The location of Katrina (latitude and longitude), sea level pressure, precipitation, and wind speeds are collected for the period August 23–31, 2005. The observed data along with the intensity changes are given in the Table 2.

### 2.3. Atmospheric Sounding and CAPE

The radiosonde datasets are provided by the University of Wyoming’ department of atmospheric sciences and is available for public at their website [23]. For each radiosonde station, the dataset listing summarizes comprehensive station information and sounding indices. The atmospheric sounding data is taken over the Gulf Coast, for the period August 23–30, 2005. From the sounding indices, the corresponding CAPE values at the equilibrium level (EL) are collected and listed in the Table 2. Using the observed CAPE, maximum vertical velocities (W_max_) are calculated from the following equations:

$${\text{W}}_{\text{max}}=2\;\surd \;(\text{CAPE})$$

where the Convective Available Potential Energy (CAPE) is defined as:

$$CAPE=\int_{el}^{lcl}g\left(\left(T_{\text{parcel}}-T_{\text{envi}}\right)/T_{envi}\right)dz$$

where, T_parcel_: temperature of the parcel,

T_envi_: temperature of the environment
g: acceleration due to gravity,
dz: differential vertical height
el: equilibrium level
lcl: level of condensation

The computed maximum vertical velocities (W_max_) over the Gulf Coast during the period August 23 –31, 2005, are given in Table 2.

## 3. Results and Discussion

**Figure 4.**
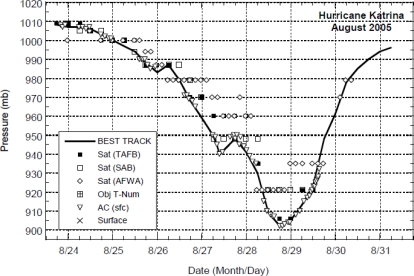
Best track positions for Hurricane Katrina, 23–30 August 2005 taken from NHC [9,10].

**Figure 5.**
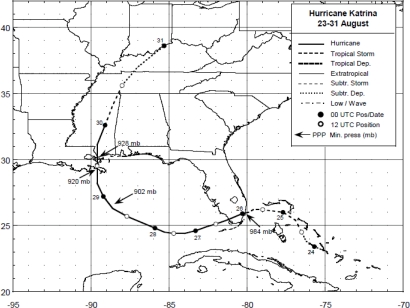
Sea Level Pressure variations of Hurricane Katrina during 23–30 August 2005. Selected pressure observations and best track minimum central pressure curve taken form NHC [9,10].

**Figure 6.**
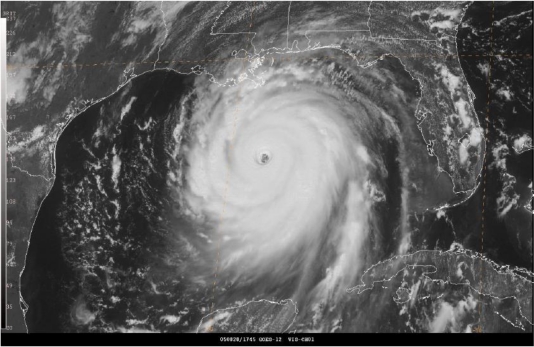
GOES-12 visible image by NHC [9,10] of Hurricane Katrina, over the central Gulf of Mexico at 1745 UTC 28 August 2005, as a category 5 near the time of its peak intensity of 150 kt.

Being spotted as a tropical depression on 23 August 2005, Hurricane Katrina began to strengthen until reaching a Category 5 on 28 August 2005. It’s winds reached peak intensity of 175 mph and the pressure fell to 902 mb before making landfall in Plaquemines Parish, Louisiana, Gulf of Mexico just South of Buras on 29th August 2005 [9,10]. The best track positions (Figure 4), and sea level pressure changes (Figure 5), and GOES satellite visible image during land fall (Figure 6) associated with Hurricane Katrina are taken from National Hurricane Center, NOAA [9,10]. At the time of landfall, the observed values of sea level pressure, wind speed, and precipitation are 927 mb, 55.8 m/s, and 10” respectively.

**Figure 7.**
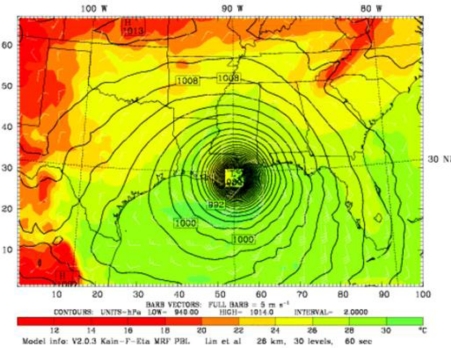
Modeling simulations of Sea Level Pressure on 28 August 2005 of 930 mb, at the time of Hurricane Katrina Land Fall.

**Figure 8.**
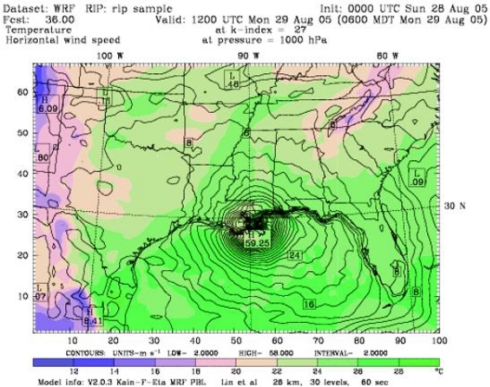
Modeling simulations of Wind Velocity of hurricane Katrina with a peak intensity of 59 m/s (175 mph), at the time of Land Fall on 28 August 2005.

**Figure 9.**
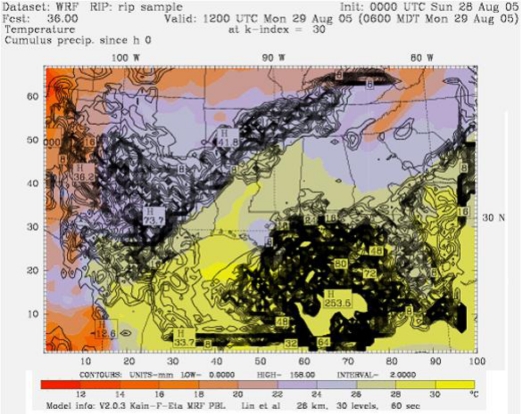
Modeling simulations of cumulative precipitation of hurricane Katrina with a maximum of 253 mm, at the time of Land Fall on 28 August 2005.

The WRF model simulations of Hurricane Katrina showed sea surface temperatures of around 34 °C over a larger area of Gulf of Mexico. The model simulations of sea level pressure, wind velocity, and cumulative precipitation at the time of landfall are shown in Figures 7, 8, and 9.

The simulated sea level pressure (930 mb), wind speed (59 ms-1), and precipitation (9.9” / 253 mm) data output were in good agreement with the observational data. Precipitation value was within the range of the rainfall forecast. Further, the model prediction revealed a storm surge of 18 ft, and flooding along with widespread thunderstorms, tornadoes.

The track and intensity changes were close to the observational data recorded by NOAA National Hurricane Center [9,10]. Reddy *et al.* [24,25] adopted numerical models (NCUR MM5, and WRF) to simulate the surface features, intensity change and track forecasting of land falling hurricanes, over the Gulf of Mexico. This study is corroborating to support the above modeling investigations including intensity change maximum sustaining winds and precipitation.

**Figure 10.**
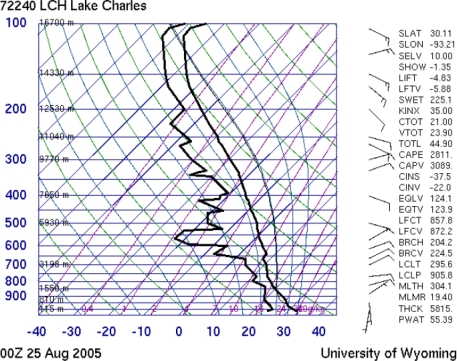
The atmospheric soundings over the Lake Charles station on 25 August 2005, from the data provided by the University of Wyoming’ department of atmospheric sciences [23]. The observed sounding indices showed a maximum value of 2811 J/kg for the CAPE at the equilibrium level (EL) about three days before the hurricane Katrina Landfall.

**Figure 11.**
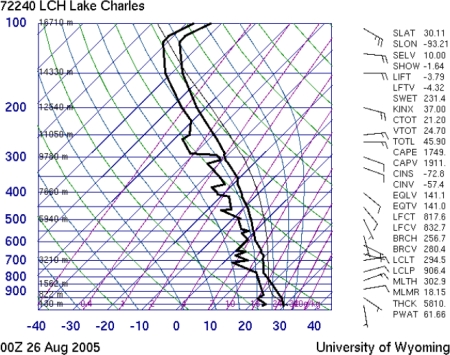
The atmospheric soundings over the Lake Charles station on 26 August 2005, from the data provided by the University of Wyoming’ department of atmospheric sciences [23]. The observed sounding indices showed still higher values of 1746 J/kg for the CAPE at the equilibrium level (EL) about two days before the hurricane Katrina Landfall,

On analyzing the data given in Table 2, the atmospheric soundings on the Gulf Coast showed maximum CAPE values were of the order of 1800–3000 J/kg during the period August 23–25, 2005. For the two days (25th, and 26th of August 2005) before Katrina landfall, the observed soundings are shown in Figures 10 and 11. The maximum vertical velocities of the order of 75 m/s were noticed on 25 August 2005, in association with the initiation of Katrina as tropical depression spotted on 23 August 2005, and as a category 5 hurricane on 28 August 2005.

The maximum CAPE and vertical motion values were noticed as a signature, two to three days before the pre-existence of the hurricane Katrina. The severe weather including thunderstorms, tornadoes, and heavy precipitation were associated with the above CAPE and vertical velocities before and during landfall. A multiscale numerical studies by Lou *et al.* [26], and Lu *et al.* [27], simulating land falling typhoons over Japan [28,29], have further supported the present results. Reddy *et al.* [30], and Tuluri *et al.* [31] have noticed heavy precipitation associated with maximum CAPE and vertical velocities for land falling tropical storms and hurricanes over the Gulf Coast.

The ability of the thunderstorms to grow primarily depends on environmental conditions favorable for the occurrence of deep convection and the corresponding atmospheric instability as measured by CAPE [4,5]. When the thunderstorms are initiated they can self-sustain in an unstable environment with strong wind shear and fueled by the latent heat released during condensation of moisture air drawn aloft from the boundary layer. Several numerical modeling have been used to simulate thunderstorms for understanding various dynamical and physical processes taking place within them [6,7]. Though, earlier numerical simulations suggest that CAPE has less significance on hurricane intensity, our study shows signs of intensifying storm may be utilized as an early warning and prediction for the pre-fall hurricane situation [6]. The CAPE as measured by atmospheric sounding is at the coast, provides a precursor to the environmental conditions hostile to the development of storm, while the hurricane is in the process of development over the sea far away from the coast. The hurricane development is a complicated process whose dynamics is still not understood completely, much so in the case of Katrina. We suppose that the simulated CAPE may not represent the real situation of the environmental conditions prevailing on the coast.

## 4. Conclusions

We have developed an advanced prediction and communication system (APCS) using environmental modeling (WRF model) and environmental technology (atmospheric sounding) tools and applied to hurricane Katrina. The APCS has shown large values of CAPE and vertical velocities as pre-existing landfall effects two to three days before land fall. The model simulations predicted low pressure of 930 mb, rainfall of 253 mm, storm surge of 18 ft, and flooding along with widespread thunderstorms and tornadoes. The model predictions are in good agreement with the observations. The APCS system will help to make a better emergency planning and management for early preparedness of the locals in the event of flood surge and inundation.

## Figures and Tables

**Table 1.** Details of the physics and grid configuration used in WRF (ARW) model.

Dynamics of Vertical Resolution	Primitive equation, no-hydrostatic 35 levels	

Domains	Domain 1	Domain 2

Horizontal Resolution	90 km	30 km
Grid Points	81 × 47	100 × 67
Domains of integration	126–W 53 W	105.6 W–75.6 W
	5 N–46 N	20.2 N–40.2 N

Initialization	NCEP Global analysis data; 2 way	
Radiation	Dudhia [18] scheme for short wave radiation, Rapid radiative transfer model (RRTM) for long wave radiation [17]	
Surface Processes	5 layer soil diffusion scheme [21]	
Boundary Layer	Blackadar Planetory Boundary Layer Parameterization [22]	
Radiation Scheme	Cloud-resolving radiation,	
Cumulus Scheme	Grell cumulus parameterization [15,16]	
Explicit Scheme	Simple ice mixing (Microphysics) scheme [14,20]	

**Table 2.** The observed time series (8/23/2005 to 8/31/2005) data of hurricane Katrina is taken from NHC [9,10]—Intensity level, location, wind speed, sea level pressure, and precipitation. Observed CAPE values are from the atmospheric sounding indices [23], and the maximum vertical velocity (W_max_) is computed from the observed CAPE.

**Time Series Data of Katrina**			**Observed Satellite Data**					**Observed CAPE, and Computed W**_**max**_	
Date	Intensity Level	Time	Latitude ° North	Longitude ° West	Wind Speed (knots)	Sea Level Pressure (mBars)	Precipitation inches	CAPE (J/kg)	Maximum Vertical Velocity (m/s)
8/23/2005	Tropical Depression	21Z	23.2	−75.5	30	1,007	-	1,889.01	61.5
8/24/2005	Tropical Depression	00Z	23.4	−75.7	30	1,007	-	1,913.42	61.9
	Tropical Storm	12Z	24.4	−76.6	35	1,006	5.90	1,515.57	55.1
8/25/2005	Tropical Storm	00Z	26	−77.7	45	1,000	3.07	2,811.67	75.0
	Tropical Storm	12Z	26.2	−79	55	994	1.63	1,014.24	45.0
8/26/2005	Hurricane-1	00Z	25.9	−80.3	70	983	7.40	1,749.6	59.2
	Hurricane-1	12Z	25.1	−82	75	979	7.71	1,656.01	57.6
8/27/2005	Hurricane-3	00Z	24.4	−84.7	100	942	0.33	816.18	40.4
	Hurricane-3	12Z	24.8	−85.9	100	941	6.94	1,209.98	49.2
8/28/2005	Hurricane-5	00Z	25.7	−87.7	145	909	2.16	1,900.4	61.7
	Hurricane-5	12Z	26.3	−88.6	150	902	0.44	57.74	10.7
8/29/2005	Hurricane-4	00Z	28.2	−89.6	125	913	14.02	1,070.05	46.3
	Hurricane-3	12Z	29.5	−89.6	110	923	10.05	161.98	18.0
8/30/2005	Tropical Storm	00Z	32.6	−89.1	50	961	3.52	197.49	19.9
	Tropical Depression	12Z	35.6	−88	30	985	0.71	33.61	8.2
8/31/2008	Extratropical	00Z	38.6	−85.3	30	994	-	121.32	15.6
